# Our Contemporaries

**Published:** 1912-01

**Authors:** 


### OUR CONTEMPORARIES

Dr. William J. Robinson announces the establishment of a new journal, the *Medical Review of Reviews,* to appear in January, incorporating *Therapeutic Medicine.* Eugenics, Sociology, History, Psychology, Current Biography, Poetry and Fiction touching upon medical practice, Cartoons, and an Index Medicus, will be prominent and more or less novel features. An excellent corps of editors is announced and, under Dr. Robinson's experienced and bold guidance, we look forward to something of unusual interest.

---

*Our Dumb Animals* for November 1911, is a good example of what the organ of humane societies ought to be. There is not too much sentiment, quite a good deal of matter intended simply to awaken an interest in the lower animals, and a practical getting after real cruelty, without a word in opposition to the scientific use of animals.

---

THE *Maryland Medical Journal,* October and November, 1911, contain the first two parts of a serial by Lewellys F. Barker, on Some Tendencies of Medical Education in the U. S. It is difficult to abstract so lengthy a consideration of the subject but we commend the serial to those engaged in medical teaching or otherwise especially interested.

---

"JANE," in the *Buffalo Express,* remarks that "A child specialist often fools the mother but he can't fool the baby." It may be said, even more broadly that a physician in any line of practice may fool the patient's friends and family, sometimes the patient, himself and other physicians, but he can't fool the disease. And it is the very general and humble recognition of this fact, dear lady, that makes the regular profession insist on certain regulations, particularly as to the training and character of its own members and of those that would compete with it, that seem arbitrary and ungenerous, officious and even cruel. This is why we persecute sweet ladies and kindly gentlemen who want to get people well in nice easy ways, without having knowledge, gained in disagreeable ways, of what their bodies are made of and how they act and of what disease consists. This is why we approve of vaccination, not because we sometimes get a dollar, more often fifty cents and, about ten times as often nothing at all, for performing the operation. This is why medical societies oppose certain laws and urge the passage of others, against the wishes of the people.

---

THE *Transvaal Medical Journal* is agitating proper qualification cation of midwives.

---

THE *Sei-i-kwai Medical Journal,* edited and published by the Sei-i-kwai. (Society for the Advancement of Medical Science) is one of our interesting exchanges which we will gladly put at the disposal of anyone who can read it. The first article is on the Origin of Syphilis, by Dr. J. Redondo, originally published Spanish in another exchange,*La Prensa Medica* of Habana and done in English (to use a distinctly English expression which is quite appropriate in the present instance) by Mariano K. Kison of Tokyo. Redondo cites from the Bible and various other ancient books, including a Chinese reference dating back to 2637 B. C., descriptions plainly referring to venereal diseases and probably to syphilis.

